# In Vitro Biological Activities of *Paederia grandidieri* Leaf Extracts

**DOI:** 10.3390/ijms252312960

**Published:** 2024-12-02

**Authors:** Faratiana Jenny Rasoariseheno, Nicoletta Fidanza, Elena Coccia, Dyana Jackson Ratovomanarivo, Daniela Sateriale, Lucia Abbatiello, Caterina Pagliarulo, Rosanna Filosa, Jeanne Angelphine Rasoamananjara, Marina Paolucci

**Affiliations:** 1 Ecole Doctorale Nutrition Environnement Santé (ED NES), University of Mahajanga, Rue Maréchal Joffre, Mahajanga 401, Madagascar; faratianar@yahoo.fr (F.J.R.); dyanaratovo@gmail.com (D.J.R.); angephi@yahoo.fr (J.A.R.); 2 Department of Science and Technology, University of Sannio, Via De Sanctis, 82100 Benevento, Italy; n.fidanza@studenti.unisannio.it (N.F.); elecoccia@unisannio.it (E.C.); sateriale@unisannio.it (D.S.); l.abbatiello3@studenti.unisannio.it (L.A.); caterina.pagliarulo@unisannio.it (C.P.); rosanna.filosa@unisannio.it (R.F.)

**Keywords:** *Paederia grandidieri*, leaf extracts, eco-compatibility, polyphenols, antimicrobial activity, prebiotic activity, cytotoxicity, healthcare

## Abstract

In many developing countries, human health problems are solved using local plants. Knowledge of their chemical composition and biological activities can contribute to the creation of natural-based products usefully employed in human health. In this work, we analysed *Paederia grandidieri* leaves extracted with diverse eco-compatible procedures and subjected to chemical, microbiological, and cellular compatibility assays. Fresh leaves of *P. grandidieri* were harvested in southern Madagascar, where most of the population relies on *P. grandidieri* for daily dental care. Leaves were dried and powdered. Distilled water and ethanol at 25 °C and 60 °C from 6 to 24 h were used for extraction. Polyphenol composition, antioxidant activity, and antibacterial, prebiotic, and cytotoxic properties of the extracts were analysed. The aqueous extracts contained higher levels of flavan-3-ols and flavanones, while the hydro-alcoholic extracts were richer in flavonols and flavones. The aqueous extracts showed the highest total phenolic and total flavonoid contents, and antioxidant activity. The hydro-alcoholic extracts showed antibacterial effects against *Listeria monocytogenes* and *Pseudomonas aeruginosa*, two key foodborne and environmental pathogens, while the aqueous extracts showed prebiotic effects against *Streptococcus salivarius*. The cytotoxic effects of *P. grandidieri* leaf extracts were evaluated using the MTT assay on mouse fibroblasts (L929 cell line). The most cytotoxic extract was the aqueous extract at 25 °C. Given that *P. grandidieri* is routinely employed by the southern Madagascar population with healthy and strong teeth with fewer cases of tooth decay with respect to other regions, and in light of the significant chemical and microbiological properties, we maintain that *P. grandidieri* can be a suitable candidate for the production of pro-health products for the oral cavity. More in-depth studies will ensure a broader picture of the impact of extracts, especially for possible oral use.

## 1. Introduction

Since ancient times, knowledge of plants with therapeutic properties has helped humankind treat and resolve various diseases [1]. Throughout history, medicinal plants have secured an important place in healing practices and treatment of diseases due to their significant health and socioeconomic benefits, especially in countries with high poverty rates and limited health coverage [2]. In developing countries, traditional plant-based medicine remains a cornerstone of healthcare, as many health problems are solved thanks to natural therapeutic solutions obtained using local plants [3]. With the advent of the post-antibiotic era [4], the spread of antibiotic resistance, and the emergence of new pathogens, herbal medicines have been widely reevaluated and have acquired a fundamental role in global health programmes, including in highly industrialized countries [5].

The economic and health relevance of plant-based medicines has increased worldwide, so much so that almost 50% of medicines are made using natural plant materials [6]. The demand for herbal medicines, natural health products, and plant secondary metabolites is growing rapidly worldwide, indicating that medicinal plants are globally valuable sources of new medicines [7]. The global market of herbal medicines is currently worth USD 215.4 billion in 2024 and is forecasted to be worth USD 305 billion by 2029 (https://www.marketdataforecast.com/market-reports/herbal-medicine-market, accessed on 9 July 2024).

In Madagascar, 93% of the population uses plants to relieve dental pain [8]. The southwestern region of Madagascar, called the Mahafaly region, is an arid and economically and climatically disadvantaged area. Nonetheless, it is an extremely relevant region, characterized by high biotic endemism, and is reported as one of the world’s 200 most important ecological areas [9]. Here, medicinal plants are a primary source of healthcare for most of the local population. Despite poverty that prevents access to basic oral hygiene products such as toothpaste and toothbrushes, this population generally shows signs of healthy and strong teeth with fewer cases of tooth decay than in other regions of Madagascar. A survey revealed that most of the population relies on plants for daily dental care, including *Paederia grandidieri*, also called Tamboro [10]. Different species of *Paederia* have been used in southern Madagascar for dental pain or to combat tooth decay; their leaves are chewed and rubbed on teeth and gums despite their repellent odour [11]. The traditional use of *Paederia* in oral pathologies has been mentioned several times in the literature, but very few scientific studies have been conducted to verify its effectiveness [11,12]. Ref. [13] reports the antimicrobial, analgesic, anti-inflammatory, and healing properties of *P. thouarsiana*, collected in eastern Madagascar, for oral treatments of painful dental pathologies of traumatic and/or post-avulsional origin.

The *Paederia* genus, belonging to the *Rubiaceae* family, includes several species known for their medicinal properties [14]. The traditional use of plants of the *Paederia* genus has been documented in several reviews [14,15,16,17]. *P. foetida* is widely used in traditional medicine to treat various disorders such as diarrhoea, dysentery, and intestinal infections [18]. Moreover, it showed significant improvement in experimentally induced colitis, likely due to its anti-inflammatory and antioxidant properties [19]. Further, it possesses moderate thrombolytic, cytotoxic, and antidiabetic effects [20], in addition to being gastroprotective [21]. Both *P. foetida* and *P. scandens* show various bioactivities like antinociceptive, anti-inflammatory, antidiarrheal, antitussive and antitumor activities [22]. The whole extract of the *P. foetida* plant showed no or limited antibacterial and antifungal activities [23], while the leaf extract proved to possess antibacterial activity against *Staphylococcus aureus* and *Escherichia coli* [24].

Research on the pharmacokinetics of *Paederia* spp. extracts, including absorption, bioavailability, distribution, metabolism, and excretion, is limited. However, based on general knowledge of herbal pharmacology, it can be inferred that the bioactive compounds in *Paederia*, such as flavonoids, are absorbed and metabolized like other plant-based substances. Many of the active compounds in *Paederia* are likely absorbed through the small intestine, similar to other herbal medicines; they are known to have moderate bioavailability and distribution, which can be influenced by factors like first-pass metabolism in the liver. Metabolites of these bioactive compounds are often more water-soluble, facilitating easier excretion [25]. Regarding the toxicity of *Paederia* extracts, while the plant is generally considered safe in traditional uses, excessive intake or improper use may lead to toxicity or adverse effects, particularly through liver or kidney stress, or herb–drug interactions [12,25].

The therapeutic potential of *Paederia* spp. is attributed to its rich phytochemical profile. The *Paederia* genus is recognized as containing numerous bioactive compounds such as iridoid glycosides (paederoside and scandoside), volatile oils, alkalodes (paederinine a and b), and flavonoid and non-flavonoid polyphenols [25,26]. Among the flavonoids, flavonol glycosides of kaempferol and quercetin were initially isolated and confirmed in the methanolic extract of the leaves and stems of *P. foetida* by HPLC [27]. In more recent times, other flavonol glycosides, such as guaiaverin, myricitrin, rutin, isorhamnetin, and a flavanone glycoside such as hesperidin, have been identified in the aerial parts of *P. foetida* by ultrasound-assisted extraction (UAE) coupled with Deep Eutectic Solvent [28]. In addition to flavonoid glycosides, non-flavonoid phenolic compounds such as caffeic acid, coumarin acid, and p-hydroxybenzoic acid have also been isolated from aerial parts and leaves of *P. foetida* by silica gel chromatography [29] while transferulic acid was isolated from the whole plant of *P. foetida* [30]. One hundred and twenty-seven phyto-compounds have been reported in the aerial elements and roots of *Paederia* spp. plant [25].

Despite the relevance of the *Paederia* genus in ethno-medicine, investigations on the chemical, microbiological, and biological properties of *P. grandidieri* are virtually absent, although a survey revealed that most of the Madagascar population relies on *P. grandidieri* for daily dental care [9,10]. For this reason, in this work, we analysed *P. grandidieri* leaves extracted with diverse eco-compatible procedures and subjected them to chemical, microbiological, and cellular compatibility assays.

## 2. Results

### 2.1. Total Phenolic Content, Total Flavonoid Content, and Antioxidant Activity of P. grandidieri Leaf Extracts

The *P. grandidieri* leaf extracts tested were aqueous (PG1, PG2, and PG3) and hydro-alcoholic (PG4 and PG5)(the extraction conditions are reported in Section 4. Briefly, the following conditions were used: PG1: aqueous extract at 25 °C for 24 h; PG2: aqueous extract at 60 °C for 6 h; PG3: aqueous extract at 60 °C for 24 h; PG4: hydro-alcoholic extract at 25 °C for 4 h; PG5: hydro-alcoholic extract at 25 °C for 24 h. The highest total phenolic content (TPC) concentration of *P. grandidieri* leaf extract, based on the Folin–Ciocalteau assay extracts, was shown by the PG1 extract, followed by PG3 and PG2, although the differences were not significant. PG4 and PG5 showed the lowest significant values. The highest total flavonoid content (TFC) based on the aluminium trichloride (AlCl3) assay was shown by the PG2 extract, followed by PG3, PG1, and PG5, although the differences were not significant. The lowest significant value was shown by PG4. The highest antioxidant activity, based on the DPPH assay, was shown by PG1 and PG3. The lowest significant percentage was reported in PG2, PG4, and PG5 extracts (Table 1).

### 2.2. HPLC Profile of P. grandidieri Leaf Extracts

**Figure 1:**
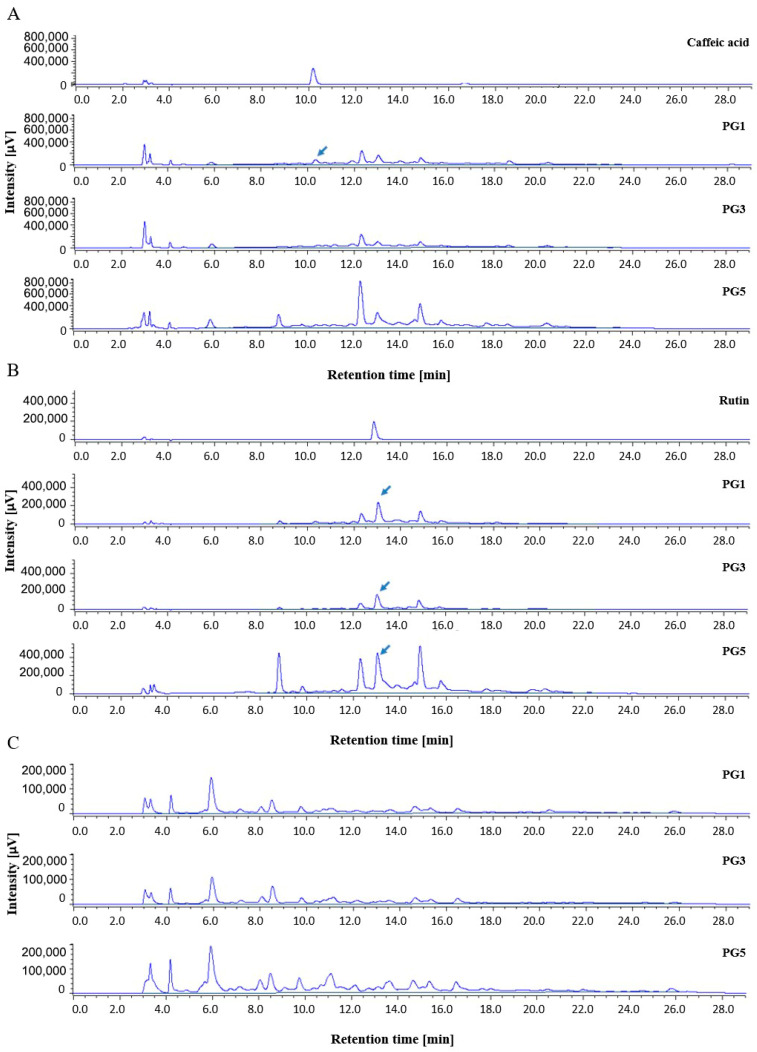
Representative chromatograms of *Paederia grandidieri* leaf extracts recorded by (**A**) UV-Vis 280 nm, (**B**) UV-Vis 360 nm, and (**C**) FDL/FP conditions. PG1: aqueous extract at 25 °C for 24 h; PG3: aqueous extract at 60 °C for 24 h; and PG5: hydro-alcoholic extract at 25 °C for 24 h. The chromatogram profiles of PG2 and PG4 are similar to those of PG3 and PG5, respectively.

Representative chromatograms of *P. grandidieri* leaf extracts are reported in Figure 1. Tentative identification of the main peaks in the chromatographic profiles was achieved using standards (caffeic acid and rutin), comparison of the retention time with the available literature [31], and UV spectra.

**Figure 2:**
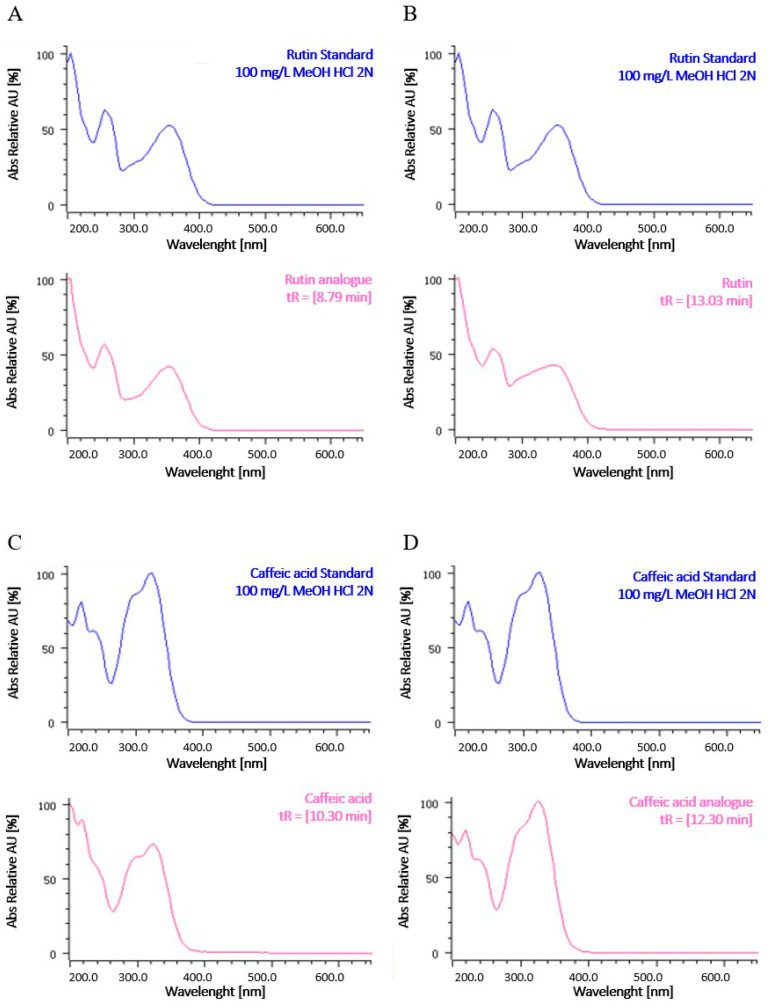
UV spectra of representative peaks at a wavelength of 280 nm. (**A**,**B**) UV spectra of compounds such as rutin analogues and rutin, respectively. Rutin was assigned based on a comparison with retention time and UV spectra of a standard reference; the rutin analogue was assigned based on UV spectra. (**C**,**D**) UV spectra of compounds such as caffeic acid and caffeic acid analogues, respectively. Caffeic acid was assigned based on a comparison with retention time and UV spectra of a standard reference; the caffeic acid analogue was assigned based on UV spectra.

UV spectra, reported in Figure 2, indicate that caffeic acid and its analogues (glycosylated compounds) and rutin and differently glycosylated forms of rutin may be present.

The TPC and flavonoids of *P. grandidieri* leaf extracts were calculated expressing the peak areas as equivalent concentrations of gallic acid, rutin, and naringin per gram of leaves (Table 2). The highest TPC yield, based on GAE, was recorded in PG1, PG3, PG4, and PG5. PG2 obtained the lowest yield. The highest amount of flavan-3-ols and flavanones evaluated by the naringin standard curve was recorded in PG1, while the highest flavonol and flavon yield, evaluated by the rutin standard curve, was recorded in PG5, followed by PG4 and PG1. PG3 and PG2 showed lower significant values.

### 2.3. In Vitro Antibacterial Activity of P. grandidieri Leaf Extracts

All aqueous and hydro-alcoholic extracts, at 10 mg/well and 20 mg/well concentrations, did not show antimicrobial activity against the following microorganisms: oral microorganisms (*Rothia dentocariosa*, *Streptococcus mutans* ATCC 25175, *Pseudomonas aeruginosa*, *Candida parapsilosis*, and *Candida albicans*); foodborne pathogens (*Escherichia coli* ATCC 25922, *Staphylococcus aureus* ATCC 25923, *Bacillus cereus* ATCC 14579, and *Salmonella enterica* ATCC 14028); environmental isolates (*Pseudomonas fluorescens*, *Rhizobium radiobacter*, *Yersinia enterocolitica*, and *Bacillus subtilis*); and microorganisms isolated from skin and mucous membranes (*Klebsiella pneumoniae*, *Staphylococcus epidermidis*, *Staphylococcus haemolyticus*, and *Candida albicans*) (Table 3 and Figures S1–S4). Interestingly, the hydro-alcoholic extracts PG4 and PG5 demonstrated significant antimicrobial activity against the foodborne pathogen *Listeria monocytogenes* and the environmental isolate *Pseudomonas aeruginosa*, with the mean diameter of the zone of inhibition (MDIZ) ranging between 20.00 ± 1.51 mm (PG4 at 10 mg/well against *L. monocytogenes*) and 32.00 ± 0.00 mm (PG5 at 20 mg/well against *P. aeruginosa*) (Table 3 and Figures S3 and S4).

The in vitro antimicrobial activity of PG4 and PG5 hydro-alcoholic extracts against *L. monocytogenes* and *P. aeruginosa* was further confirmed through quantitative assays. Minimum inhibitory concentration (MIC) and minimum bactericidal concentration (MBC) values are reported in Table 4. Both PG4 and PG5 extracts exhibited bacteriostatic effects, with MIC values of 100 mg mL^−1^ against *L. monocytogenes* and 200 mg mL^−1^ against *P. aeruginosa*, while the MBC was 200 mg mL^−1^ against *L. monocytogenes* and >200 mg mL^−1^ against *P. aeruginosa*, for both extracts.

### 2.4. In Vitro Prebiotic Effect of P. grandidieri Aqueous Extract on Streptococcus Salivarius

**Figure 3:**
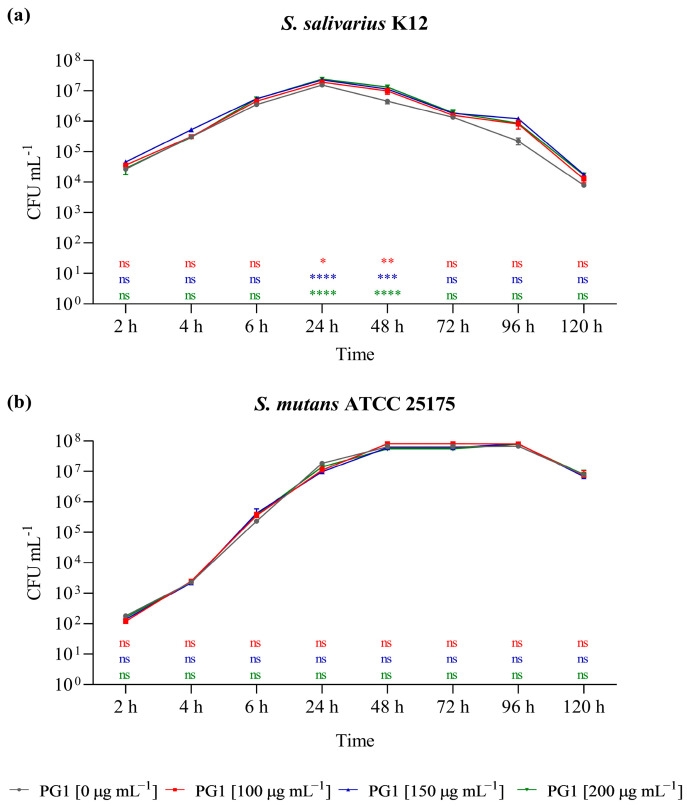
Effect of PG1 aqueous extract (25 °C for 24 h) on the survival of *Streptococcus salivarius* K12 (**a**) and *S. mutans* ATCC 25175 (**b**) in pure culture in the absence and in the presence of 100 µg µL^−1^, 150 µg µL^−1^, and 200 µg µL^−1^ of PG1. The experiments were performed in triplicate with independent cultures, and data were analysed with GraphPad Prism version 8.0.2 software. Statistical significance was examined by the two-way ANOVA test with Dunnett’s correction. Results are indicated as means ± SDs. Asterisks indicate statistical significance (**** *p* < 0.0001; *** *p* < 0.001; ** *p* < 0.01; * *p* < 0.05). ns: not significant.

The fitness of the selected bacterial isolates was monitored in the presence of different concentrations (0, 100, 150, 200 µg mL^−1^) of the PG1 extract (Figure 3). The PG1 extract was selected due to the highest TPC and flavonoid content based on the HPLC peak area evaluation and because it is the extract with an intermediate value of cytotoxicity. Although the extract did not have any impact on the survival of the cariogenic *S. mutans* ATCC 25175 strain, the survival of the probiotic *S. salivarius* K2 isolate increased in a dose-dependent manner. After 24 and 48 h of incubation in the presence of 100, 150, and 200 µg mL^−1^ of *P. grandidieri* aqueous extract, there was a significant increase in the number of viable cells of *S. salivarius* K2, supporting a prebiotic effect of the PG1 extract on oral strain at the tested concentrations.

### 2.5. In Vitro Cytotoxic Effects of P. grandidieri Leaf Extracts

**Figure 4:**
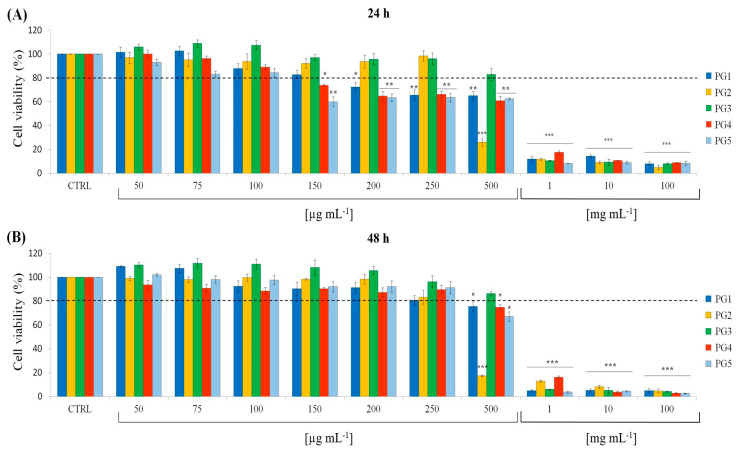
Viability of L929 cells against different concentrations of *Paederia grandidieri* extracts at 24 h (**A**) and 48 h (**B**). PG1: aqueous extract at 25 °C for 24 h; PG2: aqueous extract at 60 °C for 6 h; PG3: aqueous extract at 60 °C for 24 h; PG4: hydro-alcoholic extract at 25 °C for 4 h; and PG5: hydro-alcoholic extract at 25 °C for 24 h. Data represent mean ± SD. Significant differences from the control are defined as * *p* < 0.01, ** *p* < 0.001, and *** *p* < 0.0001. The dashed line represents the 80% cell survival threshold, corresponding to the inhibitory concentration 20 (IC20) values.

The cytotoxic effects of *P. grandidieri* leaf extracts were evaluated using the MTT assay on mouse fibroblasts (L929 cell line). Cells were treated with concentrations ranging from 50 µg mL^−1^ to 100 mg mL^−1^ for each extract for 24 and 48 h. The results were expressed as the percentage of cell viability relative to the control. Figure 4 illustrates the relationships between the extract concentrations and cell viability. At low concentrations (50–100 µg mL^−1^ for 24 h and 50–250 µg mL^−1^ for 48 h), none of the extracts showed cytotoxic effects, with cell viability remaining above 80% (dashed line). Cell viability was significantly reduced at higher concentrations (from 150 µg mL^−1^ to 100 µg mL^−1^ for 24 h and from 500 µg mL^−1^ to 100 mg mL^−1^ for 48 h), demonstrating a dose-dependent cytotoxic effect for all extracts.

Table 5 shows the IC20 values, the concentrations required to inhibit cell growth by 20%, corresponding to 80% cell survival. A cell survival of 80% is considered the accepted threshold for cell viability [32]. Notably, the PG5 extract demonstrated the highest cytotoxicity, with the lowest IC20 values, followed by PG4 > PG1 > PG2 > PG3.

## 3. Discussion

In this study, aqueous and hydro-alcoholic *P. grandidieri* leaf extracts were analysed to evaluate their antioxidant, antibacterial, prebiotic, and cytotoxic properties. Furthermore, the polyphenolic profile of the extracts was partially characterized by HPLC. Polyphenol identification was estimated through retention times, UV spectrum profiles, and comparison against HPLC standards. Although the *Paederia* genus is known for its different medicinal properties, this is the first report on *P. grandidieri*, an endemic species of southern Madagascar used by the populations for its supposed antibacterial and oral hygiene-improving properties.

The antioxidant activity of *P. grandidieri* extracts showed values ranging from 58% to 83%, which is comparable to the results reported on *P. foetida* [33]. However, total polyphenol and flavonoid values reported in this study are lower than those reported by [31] in the alcoholic extract of *P. fetida* leaves, a plant belonging to the same family as *P. grandidieri*. Such a discrepancy is likely due to the prolonged extraction times (48 h) used in *P. fetida*. Further, the antioxidant activity is influenced by several variables, such as extraction conditions (solvent, time, temperature, pH) [34] as well as the structure–activity relationship, also known as SAR, which reflects the general structure, position, and type of side groups [35]. Thus, a different polyphenol composition may affect the total antioxidant capacity of an extract. Therefore, in an attempt to obtain a more precise evaluation of the polyphenol content of *P. grandidieri* leaf extracts, we used RP-HPLC-DAD-FLD to identify and quantify subclasses of polyphenols in *P. grandidieri* leaf extracts (chromatographic fingerprint).

Chromatograms recorded at different UV and FLD values yielded different profiles depending on the sample’s composition, allowing the HPLC profile to discriminate between samples. The application of HPLC coupled to UV detection at wavelengths of 280, 320, and 360 to, respectively, identify the chemical classes of benzoic acids and flavan-3-ols (280 nm), hydroxycinnamic acids (320), and flavonols (360) is well established [36]. In particular, the chromatograms recorded at 280 nm contain information related to the overall content of phenolic acids and certain families of flavonoids (saturated and unsaturated C-ring structures) so the sum of the areas could be a reasonable index of the total phenolic content [37]. The chromatograms recorded at 360 nm give information on flavonoids with unsaturated B and C rings (flavones such as apigenin and luteolin; flavonols such as quercetin and rutin) [38]. Finally, recent studies have estimated the content of flavan-3-ols and flavanones using FLD at 280 and 330 as excitation and emission wavelengths, respectively. These wavelengths are more specific for flavonoids without an unsaturated C ring such as flavan-3 ols like catechin and flavanones like hesperetin and naringenin, while the contributions of other phenolic compounds, such as hydroxybenzoic and hydroxycinnamic acids, were found to be negligible [37]. These results have opened up new opportunities for rapid chemical and bioactive screening of polyphenol-rich extracts [39].

To compare the HPLC profile among extracts, the overlapping with intensity autoscaling was conducted. The maximum content of total polyphenols was recorded in the ethanolic extracts, and the yield of extraction was found to increase in a time-dependent manner. The qualitative analysis revealed a distinct pattern of phenolic subgroups between the aqueous and the alcoholic extracts, showing a significant influence of solvent polarity. In particular, at 25 °C, the hydro-alcoholic extracts were richer in flavonols and flavones compared to the aqueous extract. Conversely, the aqueous extract contained higher levels of flavan-3-ols and flavanones than the hydro-alcoholic extracts. These results are consistent with the literature, which reports that flavonols and flavones are less polar flavonoids and are preferentially extracted with a suitable alcohol or alcohol–water mixture, while flavan-3-ols and flavanones are extracted with water [40]. Notably, the aqueous extract at 25 °C for 24 h specifically contained caffeic acid, highlighting its solubility in water, while the hydro-alcoholic extracts revealed an analogue of rutin, indicating that the combination of alcohol and water effectively extracts flavonols with enhanced solubility. Rutin and an analogue of caffeic acid were identified in all samples. The composition of polyphenolic compounds reported in our study is consistent with that documented in the literature for other species of *Paederia* where caffeic acid and rutin were identified in the leaves, stems, and other aerial parts of *P. foetida* [25,26,28].

There are no data in the literature regarding the antibacterial activity of *P. grandidieri*, but several studies have shown that leaf extracts of *Paederia* species, such as *P. scadens* and *P. foetida*, exhibit varying degrees of antimicrobial activity [15,24,25]. Moderate antibacterial activity was reported in ethyl acetate, chloroform, and n-hexane extracts but not in the methanolic extract of *P. foetida* leaves against *Bacillus cereus*, *S. aureus, Pseudomonas aeruginosa*, and *Vibrio mimicus* [23]. Other studies have shown the antibacterial effectiveness of *Paederia* spp. leaf methanol extracts against bacteria causing diarrhoea and intestinal inflammation, such as *E. coli*, *S. aureus*, and *Mycobacterium smegmatis* [41,42]. *P. foetida* leaf extracted with ethyl acetate and ethyl alcohol inhibited *S. aureus* and *E. coli* [24]. Therefore, the literature reveals significant variability in the antibacterial effects of botanical extracts, influenced by various factors, including the botanical species, the extraction techniques, and the type of solvent, which can affect the concentration of the bioactive molecules [43,44,45]. The aqueous and hydro-alcoholic *P. grandidieri* extracts tested in this study did not show significant direct antibacterial effects against the selected oral, skin, and mucosal pathogens at the concentrations used. However, interestingly, the hydro-alcoholic extracts exhibited antibacterial activity against the foodborne pathogen *L. monocytogenes* and the environmental isolate *P. aeruginosa*. In particular, the results of the preliminary antimicrobial screening through the agar diffusion method are consistent with other studies on *Paederia* spp. extracts, including *P. foetida* and *P. scandens*, which report inhibition zones ranging from 10 to 40 mm against both Gram-positive and Gram-negative bacteria [25]. The MIC and MBC values obtained in this study were relatively high compared to those reported in the literature for other *Paederia* species. For instance, previous studies have shown that *P. foetida* and *P. scandens* extracts exhibit MIC values in the range of 2–10 mg mL^−1^ and MBC values in the range of 4–10 mg mL^−1^ [24,25,43]. In any case, the obtained results regarding the antibacterial activity of *P. grandidieri* extracts are particularly significant, because, to the best of our knowledge, there have been no previous studies reporting the antibacterial effects of *P. grandidieri* leaf extracts. The observed activity against *L. monocytogenes* and *P. aeruginosa* provides novel evidence supporting the potential of *P. grandidieri* extracts as a valuable source of antimicrobial agents, particularly against pathogens that pose serious risks to food safety and environmental health.

Regarding the prebiotic effects, there are no studies about *P. grandidieri* extracts on oral probiotics, but a recent study demonstrated how *P. scandens* ethanolic extracts contributed to modulating gut microbiota [43]. Given that numerous studies have shown the prebiotic effects of polyphenols extracted from plant sources on both intestinal and oral probiotics [45,46,47], the obtained results appear highly promising and suggest a potential indirect antibacterial effect through microbiota modulation. Indeed, the modulation of the growth of even a single probiotic strain can influence the balance of the oral microbiota in vivo, reducing the presence of pathogenic bacteria [41]. Future research aimed at confirming the ability of *P. grandidieri* extracts to beneficially modulate the oral microbiota could lead to the development of prebiotic formulations for oral health. *P. grandidieri* leaf extracts varied significantly from each other in terms of cytotoxicity. These variations could be attributed to differences in extraction conditions, such as solvent, time, and temperature, which play a crucial role in determining the chemical composition of plant extracts, thus influencing their biological activities, including cytotoxic effects [48]. In particular, the high cytotoxicity of a polyphenolic extract indicates that the extract has high biological potency and is rich in bioactive compounds [49]. In our study, the most cytotoxic extracts were the aqueous extract at 25 °C and the hydro-alcoholic extracts. Their high cytotoxicity can be attributed to their polyphenolic content. In the aqueous extract at 25 °C, we identified caffeic acid, a well-known phenolic compound with potent biological activity that may partly explain the observed cytotoxic effects. It scavenges free radicals, reduces oxidative stress, protects cells from damage, and reduces the production of inflammatory mediators at low or moderate doses, although, at high doses, it becomes cytotoxic, inducing oxidative stress, mitochondrial dysfunction, and apoptosis in various cell types, including cancer cells [50]. The hydro-alcoholic extracts displayed a richer flavonol composition, including a rutin analogue. Like caffeic acid, rutin and rutin analogues can exhibit antioxidant properties at lower concentrations, protecting cells from oxidative damage, but at higher doses, they can induce oxidative stress and apoptosis, particularly in cancer cells [51]. Such a property is beneficial for their potential use as anti-cancer agents, but it also means that high doses can harm normal, healthy cells. Investigating the dose-dependent nature is crucial for understanding its therapeutic potential and risks. While a time-dependent increase in cytotoxicity was observed in the hydro-alcoholic extracts at 25 °C, this effect was not observed in the aqueous extracts at 60 °C. Higher temperatures could enhance the solubility of bioactive compounds leading to a higher yield of certain phenolic compounds. However, prolonged exposure to heat can lead to the degradation of heat-sensitive compounds. The aqueous extract at 60 °C for 24 h showed the lowest cytotoxicity, probably due to degradative effects, which may have reduced the activity of key bioactive compounds detected. Changes in bioactivity might be due to very slight structural alterations not detected as separate peaks by standard chromatographic methods [52]. Although no prior studies have focused on the cytotoxic effects of *P. grandidieri* leaves, research on related species within the same genus provides valuable insights. Our results align with [53], who reported cytotoxic effects of the ethanolic extract of *P. foetida* at a concentration higher than 200 µg/mL. Other studies have explored the cytotoxic properties of methanolic extracts of *P. foetida* with similar results [54,55].

## 4. Materials and Methods

### 4.1. Reagents and Solutions

Methanol and acetonitrile (HPLC-grade), ethanol, formic acid, Folin–Ciocalteu reagent, aluminium trichloride (AlCl3), DPPH (2,2-Diphenyl-1-picrylhydrazyl), and standards used for HPLC analysis (quercetin, gallic acid, rutin, naringin, and caffeic acid) were purchased from Sigma-Aldrich (Milan, Italy). Ultrapure water (18 MW cm) was obtained using a Milli-Q water purification system (Millipore, Milford, MA, USA) to prepare the mobile phase and other aqueous solutions. All samples and standard solutions were filtered through 0.22 and 0.45 µm polytetrafluoroethylene (PTFE) membrane filters.

### 4.2. Sample Collection and Extraction

**Figure 5:**
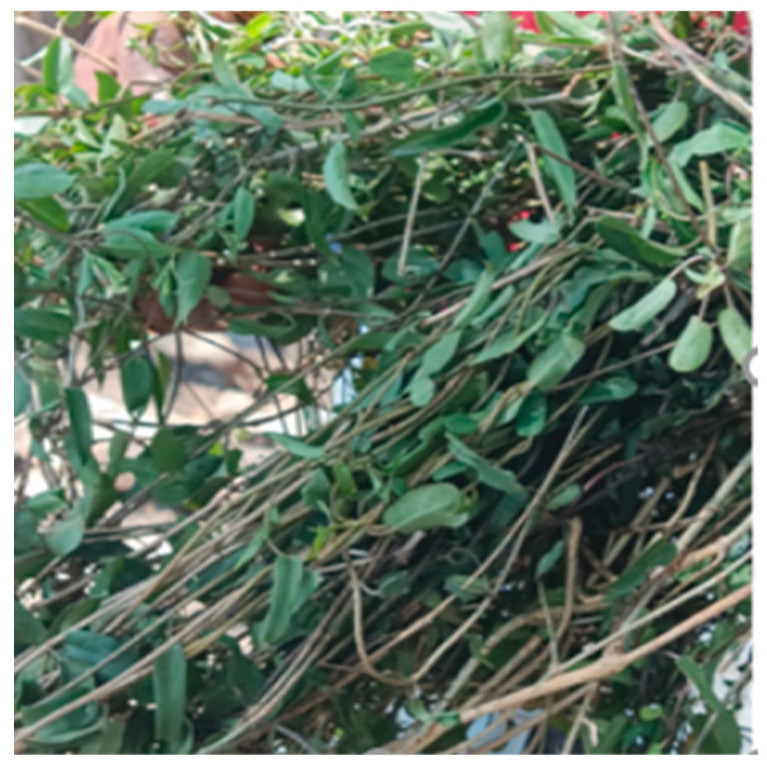
*Paederia grandidieri*.

Fresh leaves of *P. grandidieri* (Figure 5) were harvested in six districts (Toliara I and II, Ampanihy, Beloha, Tsihombe, and Ambovombe) in southern Madagascar in September and October 2023. The Botanical Survey of the University of Mahajanga Madagascar authenticated the plants.

The leaves were dried for 45 days in a cool place away from the sun according to the instructions of the University of Toliara’s botanists (Madagascar) to preserve the plant’s integrity. Thereafter, the leaves were cut into small pieces, ground into a powder using a food blender, and saved in plastic bags under vacuum. Extractions were carried out with distilled water and ethanol at different temperatures and times with continuous stirring, as reported in Table 6. The extraction mixture was filtered to separate the solid residue from the liquid phase. The filtrates were distilled under reduced pressure using a rotary evaporator and freeze-dried.

### 4.3. Total Phenolic Content

The total phenolic content (TPC) was determined by the Folin–Ciocalteu method according to [56]. Briefly, 2 mL of deionized H_2_O, 50 μL of Folin–Ciocalteu reagent, 50 μL of the filtered sample, and 100 μL of 20% Na_2_CO_3_ were used. The solution was mixed and incubated in the dark for 90 min. The absorbance was measured at 760 nm with an SP-UV1100 spectrophotometer (DLAB Scientific Co., Ltd., Beijing, China). The absorbance values were interpolated with a gallic acid standard curve, and the results were expressed as mg of Gallic Acid Equivalent (GAE)/g of dry matter. For each sample, the assay was carried out in duplicate.

### 4.4. Total Flavonoid Content

The total flavonoid content (TFC) was determined using the Dowd method [57]. Briefly, 1 mL of a 2% aluminium trichloride (AlCl_3_) water solution was mixed with the same volume of an unknown sample diluted at 1:100. After 15 min of incubation in the dark, the samples were read at 430 nm with an SP-UV1100 spectrophotometer. The standard curve was constructed with quercetin, and the concentration was expressed in mg of Quercetin Equivalents (QEs)/g of dry matter.

### 4.5. DPPH Radical Scavenging Activity

The percentage of oxidative inhibition of *P. grandidieri* extracts was evaluated using the DPPH assay as reported in [58]. Briefly, 50 μL of the extract was mixed with 2.45 mL of the DPPH methanol solution (0.004%) and incubated at RT in the dark for 30 min. The absorbance was measured at 517 nm using an SP-UV1100 spectrophotometer. Methanol was used as a blank. DPPH radical scavenging activity (RSA%) was calculated according to the following equation: RSA% = (ADPPH − A sample)/ADPPH × 100, where ADPPH is the absorbance at 517 nm of the DPPH solution and A sample is the absorbance of the sample under test.

### 4.6. HPLC/DAD/FL Analysis

Stock solutions of standard phenolic compounds were prepared in MeOH at a 4 g/L concentration and stored at −20 °C in the dark. The multicomponent standard solution was prepared to a final 1 g/L concentration by diluting the initial methanolic solutions. Working solutions in a MeOH-acidified 2N HCl solution were prepared daily by diluting the multicomponent stock solution at different concentrations ranging from 2.5 to 50 µg/mL. The *P. grandidieri* extracts were dissolved in an HCl 2N acidified methanolic solution. Dissolved samples were filtered (0.2 µm PVDF filters) and analysed by a JASCO Series 4000 compact HPLC equipped with an oven column (model CO-2060 plus), a UV/Vis Photodiode Array Detector (model MD-2018 plus), an Intelligent Fluorescence Detector (model PF-2020 plus), a liquid chromatography pump (model PU-2089 plus), an autosampler (AS-2059 plus), and the ChromNAV (version 2.0) software programme (JASCO, Easton, MD, USA). Analysis of samples was conducted in triplicate according to the conditions reported by [15,16,17,18,19,20,21,22,23,24,25,26,27,28,29,30,31]. The separation was carried out with a C18 Luna (250 mm length × 3.0 ID 5 μm particle size) column (Phenomenex, Torrance, CA, USA) at 40 °C using water with 0.1% (*v*/*v*) formic acid (mobile phase A) and acetonitrile (mobile phase B). The gradient was as follows: 0 min 90% (A), 0–17 min 40% (A), 17–22 min 40% (A), 22–28 min 90% (A), and kept at 90% (A) until the end of the run. The flow rate was 0.9 mL/min, and the injection volume was 20 µL for a total run time of 30 min.

### 4.7. Identification of Polyphenols

UV detection was carried out at wavelengths of 280 and 360 nm, and fluorescence detection (FLD) was carried out at wavelengths of 330 nm as emission after excitation at 280 nm. Specifically, the HPLC profile at 280 nm correlated with the TPC expressed as mg GAE/g [59]. The HPLC profile recorded at 360 nm correlated with flavonols (quercetin and analogues) and flavons expressed as mg Rutin Equivalent/g of the sample [60]. The HPLC profile recorded at 330 emission/280 excitation correlated with flavan-3-ols (catechins and analogues) and flavanones (naringin and analogues) [21]. Phenolic groups were quantified by comparison with standard curves of gallic acid (GA), rutin, and naringin (Figure S5). Quantifying TPC as Gallic Acid Equivalent Concentration (CGAE/mL) was performed by plotting the sum of the peak areas of the unknown sample against the slope and intercept of the GA calibration curve. A similar procedure was applied for the peak areas recorded at 360 nm and 330 emission/280 excitation, expressing the peak areas as equivalent concentrations of rutin (CRE/mL) and naringin (CNE/mL), respectively, according to [61].

The general formula to determine the CGAE is as follows:

CGAE = (∑Apeak areas at UV 280 nm − b)/m

where m is the slope of the calibration curve of GA and b is the intercept of the same calibration curve.

The general formula to determine the CRE is as follows:

CRE = (∑Apeak areas at UV 360 nm − b)/m

where m is the slope of the calibration curve of rutin and b is the intercept of the same calibration curve.

The general formula to determine the CNE is as follows:

CNE = (∑Apeak areas at FLD280–330 nm − b)/m

where m is the slope of the calibration curve of naringin carried out in the same experimental conditions (FDL 280–330 nm) and b is the intercept of the same calibration curve. The amount of each “Phenolic group” was expressed as mg “Phenolic Equivalent” per gr. of dry leaf samples.

### 4.8. Antimicrobial Assay

The in vitro antimicrobial activity of *P. grandidieri* leaf extracts was tested against oral microorganisms (*Rothia dentocariosa*, *Streptococcus mutans* ATCC 25175, *Pseudomonas aeruginosa*, *Candida parapsilosis*, and *Candida albicans*), foodborne pathogens (*Escherichia coli* ATCC 25922, *Staphylococcus aureus* ATCC 25923, *Bacillus cereus* ATCC 14579, *Salmonella enterica* ATCC 14028, and *Listeria monocytogenes*), environmental isolates (*Pseudomonas aeruginosa*, *Pseudomonas fluorescens*, *Rhizobium radiobacter*, *Yersinia enterocolitica*, and *Bacillus subtilis*), and microorganisms isolated from skin and mucous membranes (*Klebsiella pneumoniae*, *Staphylococcus epidermidis*, *Staphylococcus haemolyticus*, and *Candida albicans*); details on bacterial isolation and growth conditions are reported in [62,63]. To qualitatively assess the antimicrobial activity of extracts against selected microorganisms, an in vitro antimicrobial screening was performed using the agar well diffusion method as described by [64]. Briefly, aliquots of 200 µL of each microbial suspension, adjusted to an optical density (O.D.) of 0.5 at 600 nm, were spread on agar media, and wells with a 6 mm diameter were then created using sterile glass Pasteur pipettes, into which aliquots of extracts were dispensed. For each type of extract, two concentrations were tested: 10 and 20 mg/well. After incubation at 37 ± 2 °C for 24–48 h for all strains, the diameter of the inhibition zones formed around the discs was measured in millimetres, according to [65]. Antimicrobial activities were expressed as the mean diameter of the inhibition zones (MDIZ) produced by the extracts against the tested microorganisms. Conventional antimicrobials, including amoxicillin (Aesculapius Farmaceutici S.r.l., Brescia, Italy), gentamicin (Sigma-Aldrich S.r.l., Milano, Italy), vancomycin (Gold Biotechnology, Saint Louis, MI, USA), ampicillin (Sigma-Aldrich S.r.l., Milano, Italy), and tioconazole (Sigma-Aldrich S.r.l., Milano, Italy) were used as positive controls, while extraction buffers served as negative controls. The experiments were conducted in triplicate with independent cultures.

For the extracts that demonstrated antimicrobial activity, a quantitative in vitro assay was performed against the strains that exhibited sensitivity, to determine the minimum inhibitory concentration (MIC) and minimum bactericidal concentration (MBC), using the microdilution method as described in [66]. Briefly, a standard inoculum of 1 × 10^5^ CFU mL^−1^ was used, and extracts were prepared to achieve final concentrations of 25, 50, 100, and 200 µg µL^−1^. Samples were incubated at 37 °C for 24 h in a thermostatically controlled chamber. Conventional antimicrobials served as positive controls, while the extraction buffer was used as a negative control. The minimum inhibitory concentration (MIC) was determined as the lowest concentration of extract that inhibited microbial growth, as measured by optical density at 600 nm. Following this, aliquots were plated onto LB agar and incubated at 37 °C for 24 h. The MIC was defined as the lowest concentration of the antibacterial agent that prevents visible bacterial growth in vitro. The minimum bactericidal concentration (MBC) was determined as the lowest extract concentration which resulted in a 99% reduction in the initial bacterial count, and was assessed using the colony-forming unit (CFU) method.

### 4.9. Fitness Assay

An in vitro fitness assay was performed to quantify the effect of the *P. grandidieri* aqueous extract PG1 on the growth and survival of the probiotic strain *S. salivarius* K12 (ATCC BAA-1 024), isolated from the pharmaceutical product Bactoblis^®^ (PharmExtracta S.p.A., Pontenure, Italy). The strain was cultured under aerobic conditions at 37 °C in a Luria–Bertani (LB) medium. The fitness of the cariogenic strain *S. mutans* ATCC 25175 (LGC Standards, Middlesex, UK) in the presence of increasing concentrations of the selected extract was monitored to exclude any prebiotic effect on oral pathogens. In particular, the susceptibility of selected bacteria to different concentrations of PG1 was determined according to [58] using the dilution tube method with standard inoculums of 1 × 10^5^ CFU mL^−1^. PG1 was added to tubes at the final concentrations of 0, 100, 150, and 200 μg mL^−1^. The bacterial cultures were incubated at 37 °C in a shaking incubator (ES-20, Biosan, Riga, Latvia) at 150 rpm. To evaluate the growth and survival of probiotic strains, during the overall observation period of 120 h, aliquots of the diluted bacterial suspensions were spread on LB agar. Finally, the plates were incubated for 24 h at 37 °C to carry out the count of the viable bacterial colonies.

### 4.10. Cell Culture and Treatment

The L929 cell line (murine fibroblasts) was purchased from ATCC (American Type Culture Collection, Manassas, VA, USA) and cultured in Dulbecco’s Modified Eagle Medium (Gibco, Waltham, MA, USA) supplemented with 10% foetal bovine serum (FBS) (Merck KGaA, Darmstadt, Germany) and a 1% penicillin/streptomycin solution (10,000 U/mL/10 mg/mL; Gibco, Waltham, MA, USA). Cell cultures were maintained at 37 °C in a humidified atmosphere with 5% CO_2_ and monitored daily using an inverted microscope (Nikon, EclipseTS100, Tokyo, Japan). Subcultures were performed twice a week when 80% confluence was observed. To perform treatments, cells were seeded at a density of 5.0 × 10^3^ cells per well in 96-well plates and kept in the incubator for 24 h before stimulation. The next day, cells were treated for 24 and 48 h with increasing doses of each extract (PG1, PG2, PG3, PG4, and PG5), from 0 to 100 mg/mL. All samples were analysed in triplicate, and experiments were repeated three times with independent cell cultures.

### 4.11. In Vitro Cell Viability Studies

After stimulation, cell viability was evaluated using an MTT assay. Briefly, 20 μL of MTT solution (5 mg/mL) was added to each well. After 4h of incubation in a humidified atmosphere of 5% CO_2_ at 37 °C, the medium was removed, and the resulting formazan crystals were dissolved in 100 μL of isopropanol. Finally, the absorbance value was calculated at a wavelength of 570 nm with a spectrophotometer (Infinite F200 PRO TECAN, Grödig, Austria). The percentage of cell viability was measured according to the following equation:

% cell viability = 100 × (Abs sample/Abs control),

Abs sample was the absorbance of treated cells, and Abs control was the absorbance of untreated cells. GraphPad Prism software (GraphPad software, Inc., version 8.1.1, San Diego, CA, USA) was used to calculate the inhibitory concentration 20 (IC20) value of *P. grandidieri* extracts. Data were transformed to a log scale and normalized, setting the 0 µg/mL dose as 100% cell viability.

### 4.12. Statistical Analysis

All experiments were performed in triplicate, and the results were reported as mean ± standard deviation (SD). Data obtained were analysed by a one-way ANOVA test with Dunnett’s correction (*p* < 0.05) for a comparison with the control. Tukey’s test was used for post hoc analysis. Significant differences were defined as * *p* < 0.01, ** *p* < 0.001, and *** *p* < 0.0001. Statistical analysis was performed with GraphPad Prism software (GraphPad software, Inc., version 8.1.1, San Diego, CA, USA).

## 5. Conclusions

In the present study, aqueous and hydro-alcoholic extracts of *P. grandidieri* leaves were evaluated for their phenolic contents and biological activities from the perspective of manufacturing pro-health products for the oral cavity. In general, aqueous extracts showed higher antioxidant activity and lower cytotoxicity, as well as prebiotic properties against *S. salivarius*. The hydro-alcoholic extracts showed antibacterial effects against *L. monocytogenes* and *P. aeruginosa*. Altogether, these data suggest that *P. grandidieri* could be used as a natural compound for mouth health-promoting products. Nonetheless, these results must be interpreted cautiously since phytochemical analysis revealed that solvent, temperature, and extraction time resulted in different chemical compositions and biological activities. More extensive variations in extraction conditions should be explored in the future. Moreover, the in vitro approach has obvious disadvantages due to the difficulty in imitating the complexity of the body’s organs and systems. In the future, the obtained results will be validated using an in vivo approach and clinical studies after in vitro studies with additional human cell lines. Both in vitro studies and in vivo studies are indeed necessary for a complete applicability of the results and will provide a broader picture of the impact of the extracts, especially for possible oral use.

## Figures and Tables

**Table 1:** Total phenolic content (TPC), total flavonoid content (TFC), and % of oxidative inhibition of *Paederia grandidieri* leaf extracts. TPC and TFC are expressed as milligram equivalents of gallic acid and quercetin, respectively, per gram of dry matter. PG1: aqueous extract at 25 °C for 24 h; PG2: aqueous extract at 60 °C for 6 h; PG3: aqueous extract at 60 °C for 24 h; PG4: hydro-alcoholic extract at 25 °C for 4 h; PG5: hydro-alcoholic extract at 25 °C for 24 h.

Extract	TPC (mg GAE g^−1^)	TFC (mg QuercetinE g^−1^)	% Oxidative Inhibition
PG1	40.58 ± 4.25 ^a^	30.07 ± 1.09 ^a^	83.95 ± 0.87 ^a^
PG2	33.05 ± 5.31 ^a^	32.06 ± 0.29 ^a^	71.53 ± 1.56 ^b^
PG3	37.19 ± 2.66 ^a^	31.76 ± 0.99 ^a^	77.62 ± 4.20 ^a^
PG4	21.62 ± 2.39 ^b^	19.52 ± 1.41 ^b^	58.86 ± 4.36 ^c^
PG5	23.23 ± 0.74 ^b^	26.63 ± 3.19 ^a^	59.09 ± 1.75 ^c^

The values are indicated as mean ± SD. Letters indicate statistical significance.

**Table 2:** Total phenolic content (TPC) and flavonoids (mg/g dry vegetal product) of *Paederia grandidieri* leaf extracts. PG1: aqueous extract at 25 °C for 24 h; PG2: aqueous extract at 60 °C for 6 h; PG3: aqueous extract at 60 °C for 24 h; PG4: hydro-alcoholic extract at 25 °C for 4 h; and PG5: hydro-alcoholic extract at 25 °C for 24 h.

	TPC	Flavonoids	
	Total Phenolic Content (CGAE) (Evaluated at 280 nm)	Flavan-3-ols and Flavanones (C_NE_) (Evaluated at FDL 280–330 nm)	Flavonols and Flavones (C_RE_) (Evaluated at 360 nm)
PG1	15.81 ± 1.28 ^a^	3.55 ± 0.15 ^a^	6.61 ± 0.40 ^c^
PG2	5.33 ± 0.37 ^b^	2.34 ± 0.15 ^c^	2.11 ± 0.2 ^e^
PG3	14.05 ± 1.14 ^a^	2.51 ± 0.39 ^c^	5.31 ± 0.58 ^d^
PG4	15.51 ± 0.96 ^a^	1.29 ± 0.30 ^d^	7.48 ± 0.28 ^b^
PG5	17.14 ± 1.57 ^a^	3.09 ± 0.16 ^b^	8.77 ± 0.58 ^a^

The values are indicated as mean ± SD. Letters indicate statistical significance. FDL = fluorescence detection.

**Table 3:** In vitro antimicrobial activity of *Paederia grandidieri* leaf extracts evaluated by the agar well diffusion method. MDIZ, mean diameter of inhibition zone; PG1, aqueous extract at 25 °C for 24 h; PG2, aqueous extract at 60 °C for 6 h; PG3, aqueous extract at 60 °C for 24 h; PG4, hydro-alcoholic extract at 25 °C for 4 h; PG5, hydro-alcoholic extract at 25 °C for 24 h; AMX, amoxicillin; GNT, gentamicin; TCZ, tioconazole; VNC, vancomycin; AMP, ampicillin.

Isolation Source	Microorganism	Tested Concentration	MDIZ (mm)					
			PG1	PG2	PG3	PG4	PG5	Positive Control
Oral cavity	*S. mutans* ATCC 25175	10 mg/well	0.00 ± 0.00 **** ^a^	0.00 ± 0.00 **** ^a^	0.00 ± 0.00 **** ^a^	0.00 ± 0.00 **** ^a^	0.00 ± 0.00 **** ^a^	AMX 0.5 mg/well 37.67 ± 0.47 ^b^
		20 mg/well	0.00 ± 0.00 **** ^a^	0.00 ± 0.00 **** ^a^	0.00 ± 0.00 **** ^a^	0.00 ± 0.00 **** ^a^	0.00 ± 0.00 **** ^a^	
	*R. dentocariosa*	10 mg/well	0.00 ± 0.00 **** ^a^	0.00 ± 0.00 **** ^a^	0.00 ± 0.00 **** ^a^	0.00 ± 0.00 **** ^a^	0.00 ± 0.00 **** ^a^	AMX 0.5 mg/well 12.57 ± 0.47 ^b^
		20 mg/well	0.00 ± 0.00 **** ^a^	0.00 ± 0.00 **** ^a^	0.00 ± 0.00 **** ^a^	0.00 ± 0.00 **** ^a^	0.00 ± 0.00 **** ^a^	
	*P. aeruginosa*	10 mg/well	0.00 ± 0.00 **** ^a^	0.00 ± 0.00 **** ^a^	0.00 ± 0.00 **** ^a^	0.00 ± 0.00 **** ^a^	0.00 ± 0.00 **** ^a^	GNT 0.5 mg/well 24.00 ± 0.98 ^b^
		20 mg/well	0.00 ± 0.00 **** ^a^	0.00 ± 0.00 **** ^a^	0.00 ± 0.00 **** ^a^	0.00 ± 0.00 **** ^a^	0.00 ± 0.00 **** ^a^	
	*C. albicans*	10 mg/well	0.00 ± 0.00 **** ^a^	0.00 ± 0.00 **** ^a^	0.00 ± 0.00 **** ^a^	0.00 ± 0.00 **** ^a^	0.00 ± 0.00 **** ^a^	TCZ 0.2 mg/well 25.33 ± 0.47 ^b^
		20 mg/well	0.00 ± 0.00 **** ^a^	0.00 ± 0.00 **** ^a^	0.00 ± 0.00 **** ^a^	0.00 ± 0.00 **** ^a^	0.00 ± 0.00 **** ^a^	
	*C. parapsilosis*	10 mg/well	0.00 ± 0.00 **** ^a^	0.00 ± 0.00 **** ^a^	0.00 ± 0.00 **** ^a^	0.00 ± 0.00 **** ^a^	0.00 ± 0.00 **** ^a^	TCZ 0.2 mg/well 27.00 ± 1.00 ^b^
		20 mg/well	0.00 ± 0.00 **** ^a^	0.00 ± 0.00 **** ^a^	0.00 ± 0.00 **** ^a^	0.00 ± 0.00 **** ^a^	0.00 ± 0.00 **** ^a^	
Food	*E. coli* ATCC 25922	10 mg/well	0.00 ± 0.00 **** ^a^	0.00 ± 0.00 **** ^a^	0.00 ± 0.00 **** ^a^	0.00 ± 0.00 **** ^a^	0.00 ± 0.00 **** ^a^	GNT 0.6 mg/well 26.00 ± 1.00 ^b^
		20 mg/well	0.00 ± 0.00 **** ^a^	0.00 ± 0.00 **** ^a^	0.00 ± 0.00 **** ^a^	0.00 ± 0.00 **** ^a^	0.00 ± 0.00 **** ^a^	
	*S. aureus* ATCC 25923	10 mg/well	0.00 ± 0.00 **** ^a^	0.00 ± 0.00 **** ^a^	0.00 ± 0.00 **** ^a^	0.00 ± 0.00 **** ^a^	0.00 ± 0.00 **** ^a^	VNC 0.4 mg/well 22.33 ± 0.47 ^b^
		20 mg/well	0.00 ± 0.00 **** ^a^	0.00 ± 0.00 **** ^a^	0.00 ± 0.00 **** ^a^	0.00 ± 0.00 **** ^a^	0.00 ± 0.00 **** ^a^	
	*B. cereus* ATCC 14579	10 mg/well	0.00 ± 0.00 **** ^a^	0.00 ± 0.00 **** ^a^	0.00 ± 0.00 **** ^a^	0.00 ± 0.00 **** ^a^	0.00 ± 0.00 **** ^a^	AMX 0.5 mg/well 14.00 ± 1.00 ^b^
		20 mg/well	0.00 ± 0.00 **** ^a^	0.00 ± 0.00 **** ^a^	0.00 ± 0.00 **** ^a^	0.00 ± 0.00 **** ^a^	0.00 ± 0.00 **** ^a^	
	*S. enterica* ATCC 14028	10 mg/well	0.00 ± 0.00 **** ^a^	0.00 ± 0.00 **** ^a^	0.00 ± 0.00 **** ^a^	0.00 ± 0.00 **** ^a^	0.00 ± 0.00 **** ^a^	GNT 0.6 mg/well 23.50 ± 0.50 ^b^
		20 mg/well	0.00 ± 0.00 **** ^a^	0.00 ± 0.00 **** ^a^	0.00 ± 0.00 **** ^a^	0.00 ± 0.00 **** ^a^	0.00 ± 0.00 **** ^a^	
	*L. monocytogenes*	10 mg/well	0.00 ± 0.00 **** ^a^	0.00 ± 0.00 **** ^a^	0.00 ± 0.00 **** ^a^	20.00 ± 1.50 **** ^b^	20.50 ± 0.50 **** ^b^	AMP 0.3 mg/well 11.50 ± 0.71 ^d^
		20 mg/well	0.00 ± 0.00 **** ^a^	0.00 ± 0.00 **** ^a^	0.00 ± 0.00 **** ^a^	23.50 ± 0.50 **** ^c^	25.00 ± 0.00 **** ^c^	
Skin and mucous membranes	*K. pneumoniae*	10 mg/well	0.00 ± 0.00 **** ^a^	0.00 ± 0.00 **** ^a^	0.00 ± 0.00 **** ^a^	0.00 ± 0.00 **** ^a^	0.00 ± 0.00 **** ^a^	GNT 1.2 mg/well 23.50 ± 0.71 ^b^
		20 mg/well	0.00 ± 0.00 **** ^a^	0.00 ± 0.00 **** ^a^	0.00 ± 0.00 **** ^a^	0.00 ± 0.00 **** ^a^	0.00 ± 0.00 **** ^a^	
	*S. epidermidis*	10 mg/well	0.00 ± 0.00 **** ^a^	0.00 ± 0.00 **** ^a^	0.00 ± 0.00 **** ^a^	0.00 ± 0.00 **** ^a^	0.00 ± 0.00 **** ^a^	VNC 0.4 mg/well 23.50 ± 0.71 ^b^
		20 mg/well	0.00 ± 0.00 **** ^a^	0.00 ± 0.00 **** ^a^	0.00 ± 0.00 **** ^a^	0.00 ± 0.00 **** ^a^	0.00 ± 0.00 **** ^a^	
	*S. haemolyticus*	10 mg/well	0.00 ± 0.00 **** ^a^	0.00 ± 0.00 **** ^a^	0.00 ± 0.00 **** ^a^	0.00 ± 0.00 **** ^a^	0.00 ± 0.00 **** ^a^	VNC 0.4 mg/well 21.50 ± 0.71 ^b^
		20 mg/well	0.00 ± 0.00 **** ^a^	0.00 ± 0.00 **** ^a^	0.00 ± 0.00 **** ^a^	0.00 ± 0.00 **** ^a^	0.00 ± 0.00 **** ^a^	
	*C. albicans*	10 mg/well	0.00 ± 0.00 **** ^a^	0.00 ± 0.00 **** ^a^	0.00 ± 0.00 **** ^a^	0.00 ± 0.00 **** ^a^	0.00 ± 0.00 **** ^a^	TCZ 0.2 mg/well 24.50 ± 1.50 ^b^
		20 mg/well	0.00 ± 0.00 **** ^a^	0.00 ± 0.00 **** ^a^	0.00 ± 0.00 **** ^a^	0.00 ± 0.00 **** ^a^	0.00 ± 0.00 **** ^a^	
Environment	*P. aeruginosa*	10 mg/well	0.00 ± 0.00 **** ^a^	0.00 ± 0.00 **** ^a^	0.00 ± 0.00 **** ^a^	25.00 ± 0.00 ^b^	25.50 ± 0.71 ^b^	GNT 0.25 mg/well 25.33 ± 0.47 ^b^
		20 mg/well	0.00 ± 0.00 **** ^a^	0.00 ± 0.00 **** ^a^	0.00 ± 0.00 **** ^a^	30.00 ± 0.00 **** ^c^	32.00 ± 0.00 **** ^c^	
	*P. fluorescens*	10 mg/well	0.00 ± 0.00 **** ^a^	0.00 ± 0.00 **** ^a^	0.00 ± 0.00 **** ^a^	0.00 ± 0.00 **** ^a^	0.00 ± 0.00 **** ^a^	GNT 0.25 mg/well 19.50 ± 2.50 ^b^
		20 mg/well	0.00 ± 0.00 **** ^a^	0.00 ± 0.00 **** ^a^	0.00 ± 0.00 **** ^a^	0.00 ± 0.00 **** ^a^	0.00 ± 0.00 **** ^a^	
	*R. radiobacter*	10 mg/well	0.00 ± 0.00 **** ^a^	0.00 ± 0.00 **** ^a^	0.00 ± 0.00 **** ^a^	0.00 ± 0.00 **** ^a^	0.00 ± 0.00 **** ^a^	GNT 0.25 mg/well 27.50 ± 2.50 ^b^
		20 mg/well	0.00 ± 0.00 **** ^a^	0.00 ± 0.00 **** ^a^	0.00 ± 0.00 **** ^a^	0.00 ± 0.00 **** ^a^	0.00 ± 0.00 **** ^a^	
	*Y. enterocolitica*	10 mg/well	0.00 ± 0.00 **** ^a^	0.00 ± 0.00 **** ^a^	0.00 ± 0.00 **** ^a^	0.00 ± 0.00 **** ^a^	0.00 ± 0.00 **** ^a^	GNT 0.25 mg/well 22.50 ± 0.71 ^b^
		20 mg/well	0.00 ± 0.00 **** ^a^	0.00 ± 0.00 **** ^a^	0.00 ± 0.00 **** ^a^	0.00 ± 0.00 **** ^a^	0.00 ± 0.00 **** ^a^	
	*B. subtilis*	10 mg/well	0.00 ± 0.00 **** ^a^	0.00 ± 0.00 **** ^a^	0.00 ± 0.00 **** ^a^	0.00 ± 0.00 **** ^a^	0.00 ± 0.00 **** ^a^	AMX 0.5 mg/well 19.00 ± 1.00 ^b^
		20 mg/well	0.00 ± 0.00 **** ^a^	0.00 ± 0.00 **** ^a^	0.00 ± 0.00 **** ^a^	0.00 ± 0.00 **** ^a^	0.00 ± 0.00 **** ^a^	

The mean diameter of the inhibition zone (in mm) is reported as the mean of values obtained from assays in triplicate ± standard deviation. Statistical significance was examined by a one-way ANOVA test with Dunnett’s correction (*p* < 0.05) for comparison with the positive control and a one-way ANOVA test with Tukey’s correction (*p* < 0.05) for multiple comparisons. Asterisks indicate statistical significance with respect to the positive control (**** *p* < 0.0001); absence of asterisks indicates absence of significance. Letters (a–d) indicate statistical differences between different values; results with no significant differences receive the same letter.

**Table 4:** Quantitative evaluation of in vitro antibacterial activity of *Paederia grandidieri* hydroethanolic extracts against *Listeria monocytogenes* and *Pseudomonas aeruginosa*. PG4, hydro-alcoholic extract at 25 °C for 4 h; PG5, hydro-alcoholic extract at 25 °C for 24 h; GNT, gentamicin; AMP, ampicillin; MIC, minimum inhibitory concentration; MBC, minimum bactericidal concentration; nt, not tested.

Microorganisms	PG4 [mg mL^−1^]		PG5 [mg mL^−1^]		GNT [µg mL^−1^]		AMP [µg mL^−1^]	
	MIC	MBC	MIC	MBC	MIC	MBC	MIC	MBC
*L. monocytogenes*	100	200	100	200	nt	nt	0.5	2.5
*P. aeruginosa*	200	>200	200	>200	<0.1	0.1	nt	nt

**Table 5:** Inhibitory concentration 20 (IC20) values of *Paederia grandideri* leaf extracts at 24 and 48 h. PG1: aqueous extract at 25 °C for 24 h; PG2: aqueous extract at 60 °C for 6 h; PG3: aqueous extract at 60 °C for 24 h; PG4: hydro-alcoholic extract at 25 °C for 4 h; and PG5: hydro-alcoholic extract at 25 °C for 24 h. IC20 values represent the concentrations required to inhibit 20% of cell viability for different extracts of *P. grandidieri* at 24 and 48 h. The IC20 values are expressed in µg mL^−1^ and indicate the cytotoxic potency of each extract. Lower IC20 values correspond to higher cytotoxicity.

Extract	IC20 (24 h) (µg mL^−1^)	IC20 (48 h) (µg mL^−1^)
PG1	168 ± 1.8 ^b^	283 ± 1.6 ^c^
PG2	360 ± 2.7 ^d^	258 ± 2.1 ^c^
PG3	528 ± 3.8 ^e^	600 ± 3.2 ^e^
PG4	170 ± 2.5 ^b^	246 ± 1.9 ^c^
PG5	125 ± 2.1 ^a^	240 ± 1.7 ^c^

The values are presented as mean ± SD based on triplicate measurements. Letters indicate statistical significance.

**Table 6:** Extraction conditions of *Paederia grandidieri* leaves.

Extract	Solvent	Ratio (*w*/*v*)	Temperature (°C)	Time (h)
PG1	H_2_O	1:50	25 °C	24
PG2	H_2_O	1:50	60 °C	6
PG3	H_2_O	1:50	60 °C	24
PG4	EtOH 70%	1:10	25 °C	4
PG5	EtOH 70%	1:10	25 °C	24

## Data Availability

The raw data supporting the conclusions of this article will be made available by the authors, without undue reservation.

## Supplementary Materials

The following supporting information can be downloaded at https://www.mdpi.com/article/10.3390/ijms252312960/s1.
