# Supplementary material for: In Vitro Biological Activities of Paederia grandidieri Leaf Extracts
Source: Int J Mol Sci. 2024 Dec 2;25(23):12960. doi: 10.3390/ijms252312960 (PMC11641373; doi:10.3390/ijms252312960)
Supplement: Supplementary file 1 [file ijms-25-12960-s001.zip › Supplementary Figures.pdf]

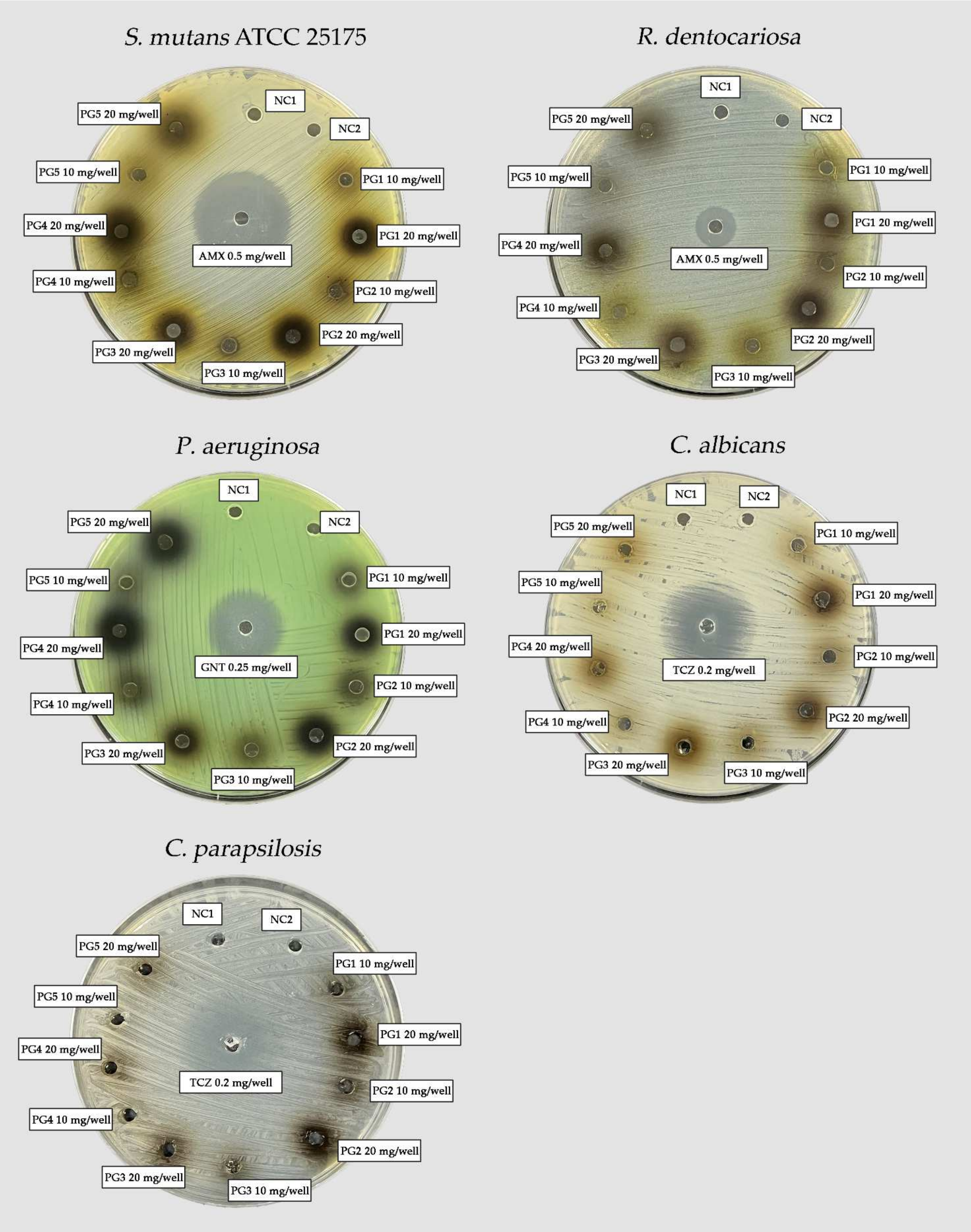

Figure S1: *In vitro* antimicrobial activity of *P. grandidieri* leaf extracts against oral microorganisms (*S. mutans* ATCC 25175; *R. dentocariosa*; *P. aeruginosa*; *C. albicans; C. parapsilosis*) evaluated by agar well diffusion method. NC1, H_2O; NC2, EtOH 70%; PG1, aqueous extract at 25°C for 24 hours; PG2, aqueous extract at 60°C for 6 hours; PG3, aqueous

extract at 60°C for 24 hours; PG4, hydro-alcoholic extract at 25°C for 4 hours; PG5, hydro-alcoholic extract at 25°C for 24 hours; AMX, amoxicillin; GNT, gentamicin; TCZ, tioconazole.

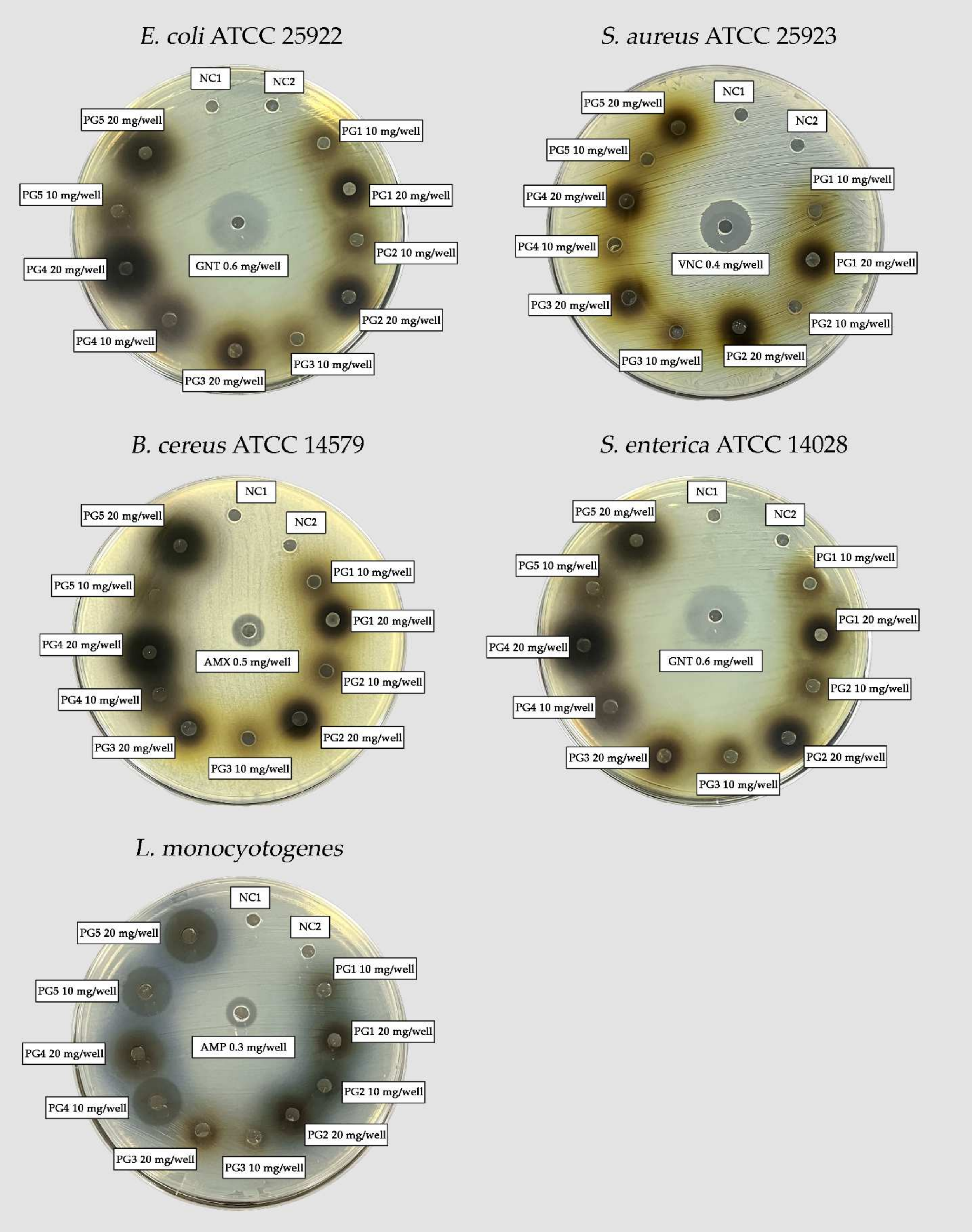

Figure S2: *In vitro* antimicrobial activity of *P. grandidieri* leaf extracts against foodborne pathogens (*E. coli* ATTC 25922; *S. aureus* ATCC 25923; *B. cereus* ATCC 14579; *S. enterica* ATCC 14028; *L. monocytogenes*) evaluated by agar well diffusion method. NC1, H_2O; NC2, EtOH

70%; PG1, aqueous extract at 25°C for 24 hours; PG2, aqueous extract at 60°C for 6 hours; PG3, aqueous extract at 60°C for 24 hours; PG4, hydro-alcoholic extract at 25°C for 4 hours; PG5, hydro-alcoholic extract at 25°C for 24 hours; AMX, amoxicillin; GNT, gentamicin; VNC, vancomycin; AMP, ampicillin.

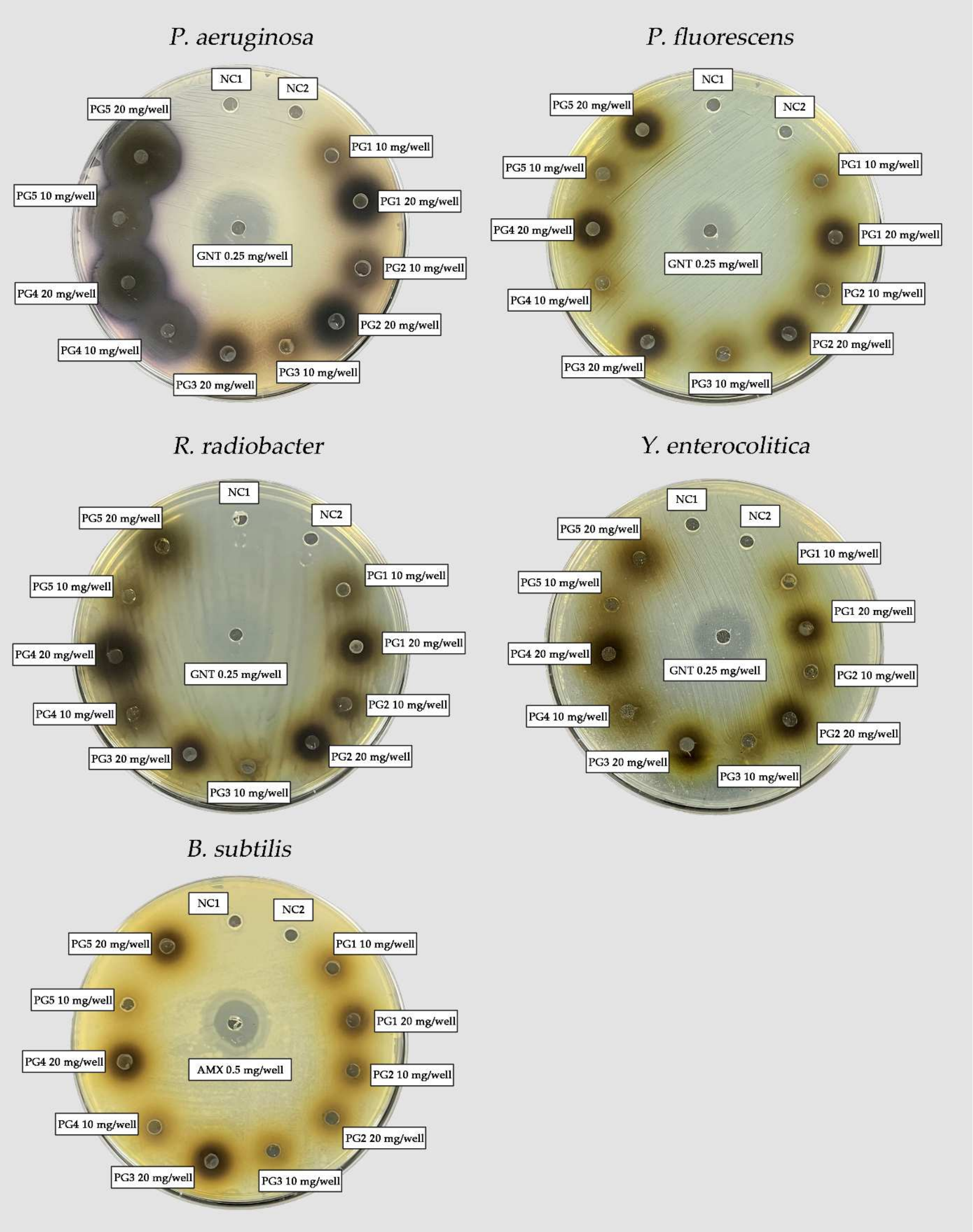

Figure S3: *In vitro* antimicrobial activity of P. *grandidieri* leaf extracts against environmental isolates (*P. aeruginosa*; *P. fluorescens*; *R. radiobacter*; *Y. enterocolitica*; *B. subtilis*) evaluated by

agar well diffusion method. NC1, H_2O; NC2, EtOH 70%; PG1, aqueous extract at 25°C for 24 hours; PG2, aqueous extract at 60°C for 6 hours; PG3, aqueous extract at 60°C for 24 hours; PG4, hydro-alcoholic extract at 25°C for 4 hours; PG5, hydro-alcoholic extract at 25°C for 24 hours; AMX, amoxicillin; GNT, gentamicin.

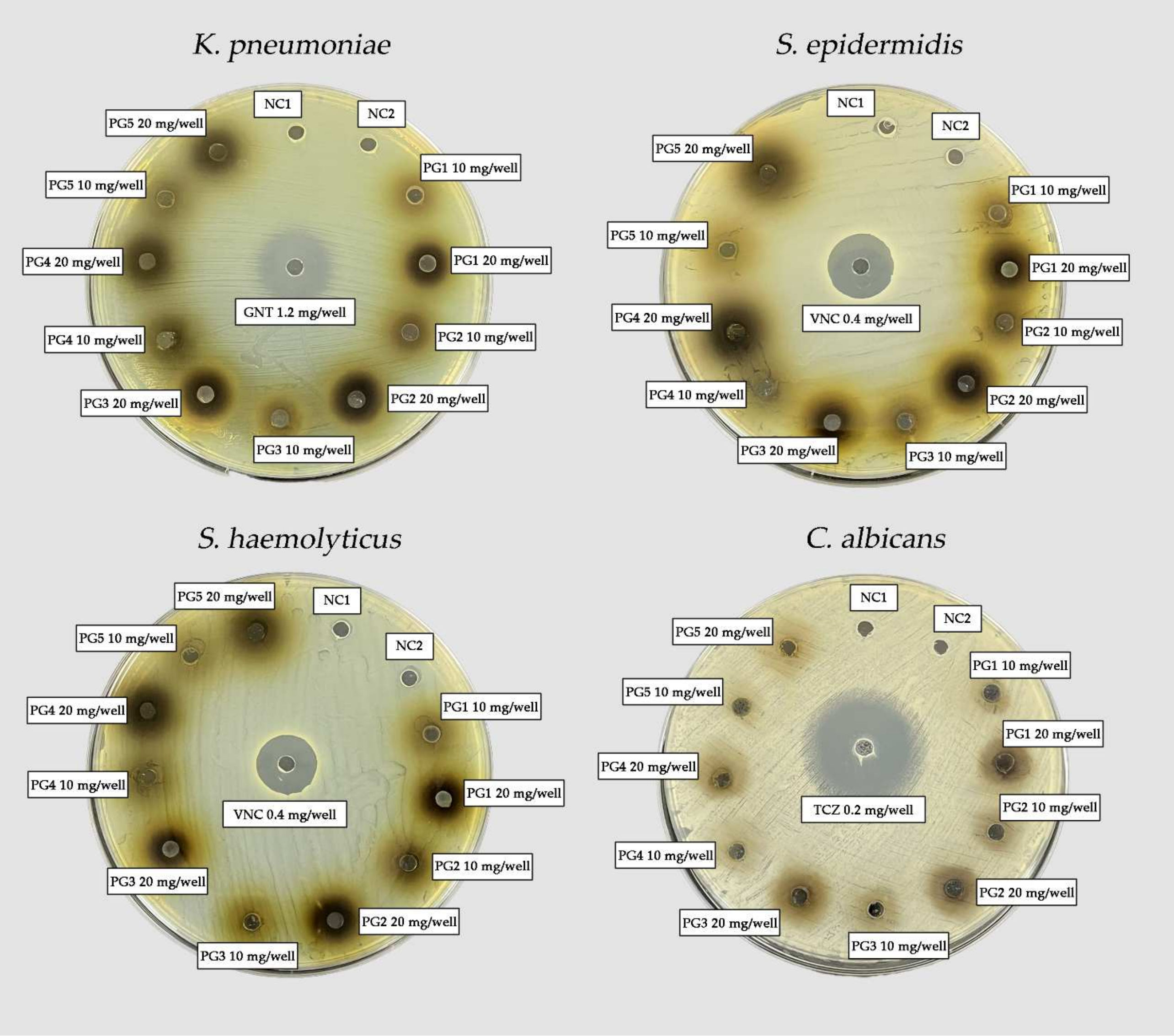

Figure S4: *In vitro* antimicrobial activity of *P. grandidieri* leaf extracts against microorganisms isolated from skin and mucous membranes (*K. pneumoniae*; *S. epidermidis*; *S. haemolyticus*; *C. albicans*) evaluated by agar well diffusion method. NC1, H_2O; NC2, EtOH 70%; PG1, aqueous extract at 25°C for 24 hours; PG2, aqueous extract at 60°C for 6 hours; PG3, aqueous extract at 60°C for 24 hours; PG4, hydro-alcoholic extract at 25°C for 4 hours; PG5, hydro-alcoholic extract at 25°C for 24 hours; GNT, gentamicin; TCZ, tioconazole; VNC, vancomycin.

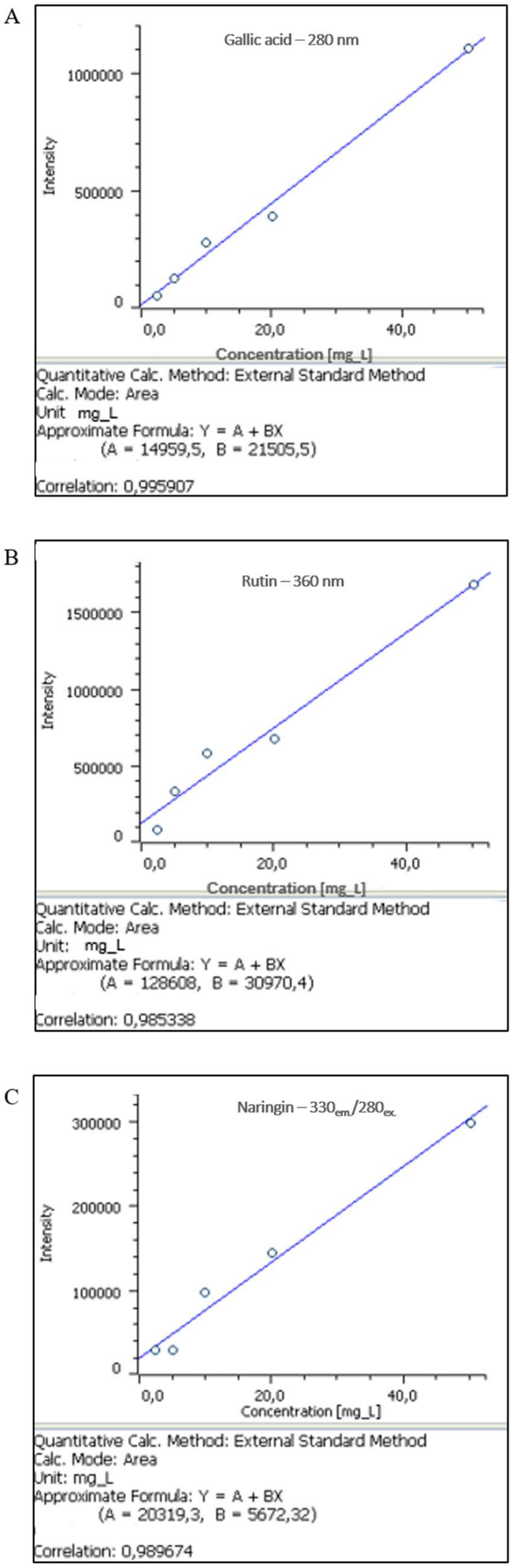

Figure S5: Standard curves of A) gallic acid, B) rutin and C) naringin at concentrations of 2.5, 5, 10, 20 and 50 mg/L.
